# Comparing Young and Old Adults’ Night Hazard Detection With Driving Simulation and on Road

**DOI:** 10.1093/geroni/igab046.1020

**Published:** 2021-12-17

**Authors:** Anne Dickerson, Juliette Leonardo

**Affiliations:** East Carolina University, Greenville, North Carolina, United States

## Abstract

While there is validity of using driving simulation as a proxy for on-road performance, few studies have examined hazard detection at night. Night driving is a self-restricting practice with little evidence demonstrating the need with healthy older adults. This study’s objective was to analyze night driving using eye-tracking technology examining differences between on-road/simulated drives and older/younger adults. A 2 (old, young) x 2 (simulator, on-road) repeated-measures design measured three roadway “hazards” of pedestrains looking at their cell phone while posed to cross the roadway. Pupil glances were recorded using outcome measures of total fixation duration, number of fixations, and time-to-first fixation for the pedestrains on-road and on a specifically designed scenario matching the on-road route. Thirty-three healthy, community-living drivers age 65+ years (N=16) and drivers age 20-40 years (N=17) completed both drives. Using non-parametric statistics, results demonstrated that night hazard detection was similar across driving conditions except for time-to-first fixation, which was faster on-road for both age groups (p<.001). At some hazard locations, there were significant differences between the two age groups, with older adults taking longer to initially see hazards. Results suggest, older adults detected hazards similarly to younger adults, especially during on-road performance, suggesting avoidance of night driving may not be necessary. Results also support using driving simulation as a proxy for on-road with night driving needing to be incorporated. Additionally, eye-tracking has the potential for research in hazard detection with emphasis on the time-to-first fixation outcomes when considering driving analysis.

